# In vivo depletion of CD25+ cells prior to LRAST increases therapeutic efficacy in a murine melanoma model

**DOI:** 10.1186/2051-1426-2-S3-P51

**Published:** 2014-11-06

**Authors:** Julia R Kovács, Peter Rose, Louis Boon, Rudolf A Hatz, Hauke Winter, Natasja K van den Engel

**Affiliations:** 1 University of Munich, Campus Grosshadern, Germany; 2 Bioceros, Netherlands

## 

Lymphodepletion, immune reconstitution and active- specific tumor cell vaccination (LRAST) have proven to benefit the activation of tumor-specific T cells in mice during homeostatic proliferation. Immunoregulatory mechanisms like the induction of regulatory T cells may counteract this beneficial effect. To further clarify the role of regulatory T cells (Treg) during LRAST, we inhibited CD25^+ ^cells in donor mice and/or in recipient mice *in vivo*, using the monoclonal anti-CD25 antibody PC61. We investigated the induction of an anti-tumor immune response by analyzing tumor growth and the induction of tumor-specific T cells following LRAST.

C57BL/6 recipient mice were pre-treated with a monoclonal anti-CD25 antibody (PC61, 250µg i.p.) or a corresponding isotype control (anti-βGal, 260µg, i.p.) twice before tumor inoculation with murine melanoma cells (D5, 5x10^4 ^cells s.c.). Three days later, lymphopenia was induced by injection of cyclophosphamide (200 mg/kg). The next day, mice were reconstituted with spleen cells from a congenic mouse strain (C57BL6-Ly5.1, 2x10^7 ^cells i.v.) either pre-treated with anti-CD25 or the isotype antibody. The effect of the anti-CD25 antibody on CD45^+^CD4^+^CD25^+^Foxp3^+ ^(Treg) levels in the peripheral blood was analyzed by flow cytometry. Tumor growth was monitored and the induction of tumor-specific T cells was analyzed by IFN-γ cytokine release assay.

Three of five (60%) of the mice treated with anti-CD25 antibody and reconstituted with spleen cells from Treg-depleted donor mice showed a prolonged survival in comparison to mice treated with isotype antibody and reconstituted with spleen cells from naïve donors. Treg-depleted hosts reconstituted with cells from donor mice treated with an isotype control showed the highest release of tumor-specific IFN-γ in tumor-draining lymph node cells. Interestingly, reconstitution with anti-CD25-depleted spleen cells significantly reduced tumor-specific IFN-γ release in tumor-draining lymph node cells.

Treg inactivation with anti-CD25 antibody prior to LRAST increases therapeutic efficacy. These results suggest that CD25 depletion of the host, but not of the donor, is advantageous for the induction of a tumor-specific immune response. Whether this treatment protocol results in a therapeutic immune response and further improves survival is currently under investigation.

